# *Stocky1*, a Novel Gene Involved in Maize Seedling Development and Cuticle Integrity

**DOI:** 10.3390/plants11070847

**Published:** 2022-03-23

**Authors:** Angelo Gaiti, Stefano Sangiorgio, Franco Faoro, Carlo Massimo Pozzi, Giuseppe Gavazzi, Salvatore Roberto Pilu

**Affiliations:** Department of Agricultural and Environmental Sciences—Production, Landscape, Agroenergy, Università degli Studi di Milano, Via G. Celoria 2, 20133 Milan, Italy; angelo.gaiti@unimi.it (A.G.); stefano.sangiorgio@unimi.it (S.S.); franco.faoro@unimi.it (F.F.); carlo.pozzi@unimi.it (C.M.P.); giuseppe.gavazzi@unimi.it (G.G.)

**Keywords:** plant cuticle, seedling development, maize cuticle

## Abstract

The cuticle is the plant’s outermost layer that covers the surfaces of aerial parts. This structure is composed of a variety of aliphatic molecules and is well-known for its protective role against biotic and abiotic stresses in plants. Mutants with a permeable cuticle show developmental defects such as organ fusions and altered seed germination and viability. In this study, we identified a novel maize mutant, *stocky1*, with unique features: lethal at the seedling stage, and showing a severely dwarfed phenotype, due to a defective cuticle. For the first time, the mutant was tentatively mapped to chromosome 5, bin 5.04. The mutant phenotype investigated in this work has the potential to contribute to the elucidation of the role of the cuticle during plant development. The possibility of controlling this trait is of relevance in the context of climate change, as it may contribute to tolerance to abiotic stresses.

## 1. Introduction

### 1.1. Embryogenesis and Seedling Architecture

In maize (*Zea mays*), soon after pollination, the embryo develops from the zygote and acquires a bilateral symmetry, and the first cell types start differentiating [1]. The two future meristems, the shoot apical meristem (SAM) and the root apical meristem (RAM), are specified, while being surrounded by the coleoptile and the coleorhiza. Shortly after, a shield-shaped organ, the scutellum (a cotyledon), originates from the embryo [2]. The development of the embryo and the endosperm occurs rapidly from single cells into highly differentiated tissues during the first 15 days after pollination (DAP) [3]. The seed development proceeds into the filling stage and subsequently into the dehydration phase, and this takes around 60 days to complete.

The architecture of the seedling is established during embryogenesis: the first shoot internode is formed just above the scutellar node. Five or more short internodes develop above the coleoptilar node, together with the respective leaf primordia. The leaf primordia are arranged in a conical, telescopic order, encapsulated in an external protective structure, the scutellum. Once the embryo bauplan is completed, germination occurs. During this phase, the plant’s development witnesses many catabolic events until the start of autotrophic growth [4]. During germination the coleoptile elongates, piercing the soil, while protecting the developing leaflets during their initial, delicate phase of growth. Subsequently, the coleoptile opens and deteriorates, allowing the leaves to emerge. During this process, a switch from developmental to germinative growth occurs. This coordinated process requires a complex and interconnected network coordinated at the molecular level [5].

The occurrence of the phases described above may be less strict in other species. In Arabidopsis, for example, some mutants, identified based on abnormal vegetative growth, are also defective in embryogenesis, suggesting that the genes necessary for post-embryonic development are also active late in embryogenesis [6].

### 1.2. The Cuticle Role

The cuticle is a hydrophobic layer that covers plant aerial organs. The cuticle is responsible for many functions in plants: it prevents dehydration, limits gaseous exchanges, protects the plant from extreme temperatures, UV (ultraviolet) adiation and pathogens, provides mechanical strength, and prevents organ fusion during development [7]. Cuticle synthesis is initiated during embryogenesis, and its deposition is coordinated with plant growth [7,8]. The cuticle is composed of several layers, the innermost of which, the cutin, is in direct contact with the epidermal cell wall. Waxes are either interspersed in the cutin or are in direct contact with the environment [9]. The building blocks that compose the cuticle are fatty acid-derived compounds, which are synthetized in the endoplasmic reticulum of epidermal cells before being exported to the epidermis face in contact with the external environment [10]. The gene network which regulates cuticle formation has been elucidated in Arabidopsis and maize [7], and it involves transcription factors of the AP2/ERF and MYB family [11,12,13]. 

Wild-type maize’s most juvenile leaves have epicuticular waxes, and they later develop a glossy appearance [12]. Mutants’ seedlings where the juvenile wax synthesis does not occur are easily recognized because of the glossy phenotype of the first few leaves [14]. Several of the genes responsible for the glossy phenotype have been cloned and, in some cases, extensively characterized [15]. 

The *fused leaves 1* mutant (*fdl1*) has been molecularly characterized [16], and the mechanisms by which it interacts with another gene, *ADHERENT1* (*AD1*), were elucidated [11,17]. *ad1* mutant shows organ fusion defects during both juvenile and male reproductive development [17]. The fused *fdl1* phenotype is caused by an altered cuticle deposition in young seedlings and by the irregular epicuticular wax deposition on the young leaf’s epidermis. The mutation responsible for *fdl1* is caused by a transposon insertion into an MYB gene (*ZmMYB94*) [16], while AD1 encodes a 3-ketoacyl-CoA synthase (KCS) [18] involved in the cuticular wax biosynthesis [11]. The organ adherence in *ad1* mutants is caused by epidermal fusions among and between different tissues and organs in both juvenile and reproductive stages. This suggests that AD1 plays an important role in maintaining proper organ separation. It is now known that many genes involved in cuticle formation are interplaying in maize [19,20].

### 1.3. Mutants for the Study of Seedling Development in Maize

In maize, a class of mutants designated *des* (defective seedling), showing disrupted seedling morphogenesis, represents a good candidate for the isolation of genes involved in the formation of the plant body pattern during embryogenesis. A series of *des* mutants, identified on the basis of abnormal vegetative growth, has been previously described [21,22,23,24]. 

In this paper, we isolated six mutants defective in seedlings’ development. We report a preliminary characterization of one of them, defective seedling*-19 (des*-19). The mutant des*-19 is named *stocky1* from now on. The mutant shows unique features because it is lethal at the seedling stage, and it has a severely dwarfed phenotype due to a defective cuticle. For the first time, the mutant was tentatively mapped to chromosome 5, bin 5.04. The mutant phenotype investigated in this work has the potential to contribute to the elucidation of the role of wax deposition during plant development. The possibility to control this trait is of relevance in the context of climate change, as it may contribute to tolerance to abiotic stresses [25].

## 2. Results

### 2.1. Origin and Isolation of Defective Seedlings “des” Mutants

**Figure 1:**
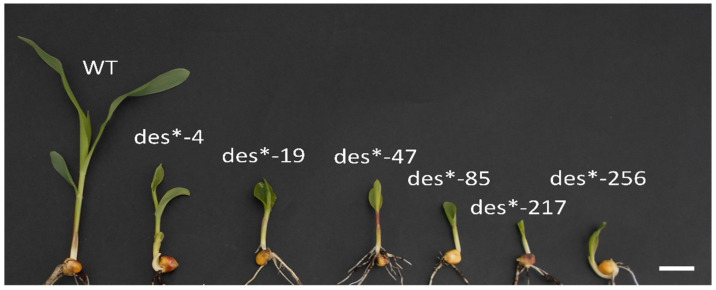
Phenotype of *des* mutant seedlings: pictures of wild type (WT) and mutant seedlings obtained from the EMS mutagenized population at 10 days after sowing (DAS): WT, des*-4, des*-19/stocky1, des*-47, des*-85, des*-217, des*-256. Scale bar: 1 cm. The mutants show a dwarf phenotype, and are delayed in the first phases of seedling development.

A population of maize was mutagenized in 1998 by treating pollen with ethyl methanesulfonate (EMS) prior to pollination. Mutants showing developmental defects after emergence were isolated. Six independent mutants, called *defective seedlings* (des*), were identified in the M2 population (Figure 1).

Segregation analyses of the des* mutants indicated that they were inherited as a monogenic recessive trait (Table 1). A complementation analysis was then performed, and the loci proved to be non-allelic (Table 2). To ascertain the presence of gametophytic selection in des*-19, we performed a segregation analysis using seeds from the basal and distal region of the ears: no differences between the ears’ regions were found (data not shown).

### 2.2. Phenotypic Characterization of Stocky1

**Figure 2:**
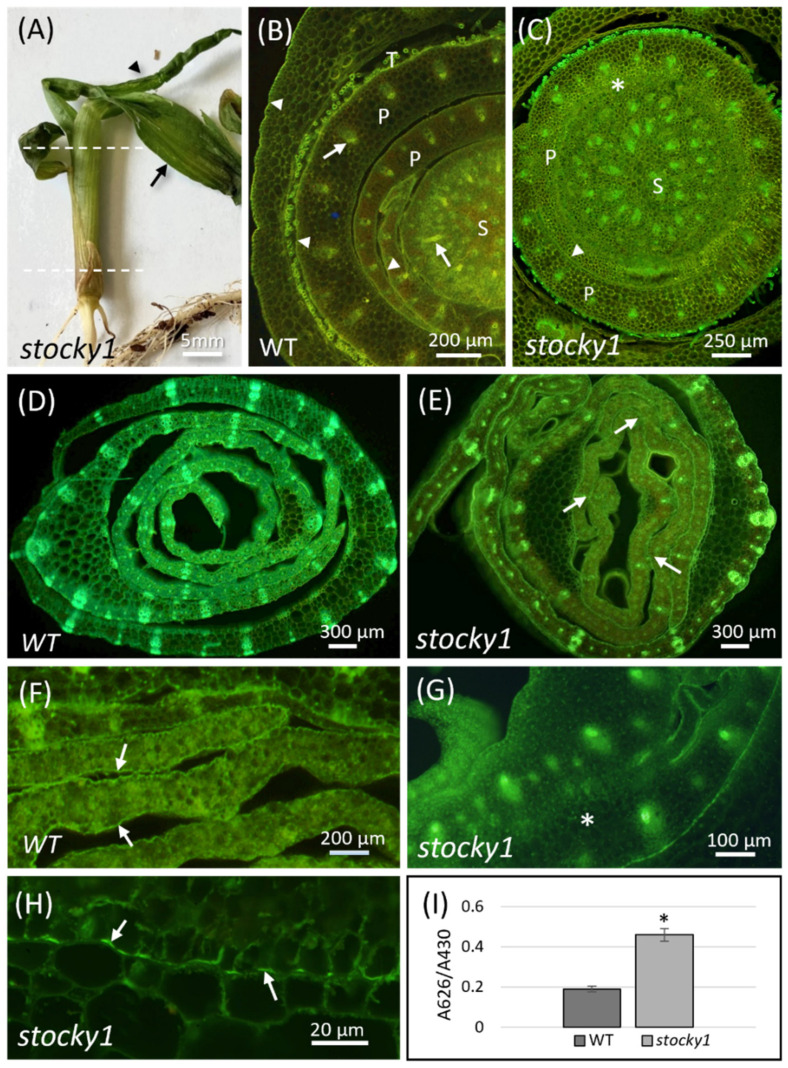
Phenotypic characterization of *stocky1* seedlings: (**A**) Representative *stocky1* seedling at 12 DAS showing the first three developing leaves; the third leaf (arrowhead) is bent, rolled and twisted, and has been released manually from the second one (arrow), which is ripped in the adhesion regions; the lower and upper dashed lines indicate, respectively, the proximal and distal part from which cross sections shown in C, E, G, H, have been cut. Cross sections shown in B, D, F, are from WT seedlings and were cut in the same regions for comparison; all sections were stained with Calcofluor White and Auramine-O, the latter specific for the cuticle, which stains in bright yellow-green when excited at about 450 nm by the fluorescence microscope. (**B**) WT proximal region showing normally developed leaf primordia [P] rolled up around the stem [S] and outlined by a bright yellow cuticle (arrowheads); leaf trichomes [T] and xylem vessels (arrows) are also visible by autofluorescence. (**C**) *stocky1* proximal region with leaf primordia [P], in some parts not completely separated from each other (asterisk) and with a less defined cuticle (arrowhead); the stem [S] is larger than in WT seedlings. (**D**) Distal part of a WT seedling with rolled and well-separated developing leaves which show a continuous cuticle, more clearly visible in the enlargement in **F** (arrows). (**E**) Distal part of a *stocky1* seedling in which developing leaves are fused together in some regions (arrows); in these regions, the cuticle of the fused leaves is not visible, as shown in the enlargement in **G** (asterisk); in other fused regions, the cuticle appears discontinuous (**H**, arrows). (**I**) TB permeability quantification in the wild type and in the *stocky1* mutant showing a higher TB uptake in the latter; error bars represent SD from three biological replicates; black asterisk indicates a statistically significant difference (* *p* < 0.01) obtained with *t*-Test.

The *stocky1* mutant was selected for further analysis because of its unique phenotype. The *stocky1* seedlings are in fact easily recognized shortly after emergence (about 10 days after sowing, DAS), since they form a cone-shaped structure which prevents seedlings’ development (Figure 2A). This structure develops either from the first or from the second developing leaves. Inside the cone, these leaves are wrapped one inside the other, and their expansion is inhibited by adhesion regions. Consequently, the shoot undergoes a rapid bending, causing extensive leaf tearing. The seedling dies at about 25–30 DAS. Cross sections of *stocky1* shoots were stained with Calcofluor White and Auramine-O to highlight cell wall and cuticle components, and were observed by epifluorescence microscopy. Leaf primordia in the WT shoot proximal region were normally developed and well-separated from each other (Figure 2B). Conversely, in the mutant, the leaf primordia were fused (Figure 2C). The difference between mutant and WT was more apparent in the distal region: here, the WT developing leaves were completely separated, as shown by the cuticle-staining Auramine-O signal (Figure 2D,F). In *stocky1* seedlings, the canonical organization was lost, and leaves’ fusions appeared in many regions where cuticle was lacking (Figure 2E,G,H). To further understand the involvement of the cuticle in the *stocky1* phenotype, the cuticle integrity was then assessed with the toluidine blue (TB) test permeability assay on seedlings after 10 DAS. The quantification of TB uptake shows a higher permeability to the dye in the mutant versus WT seedlings (Figure 2I).

### 2.3. Role of Hormones

**Figure 3:**
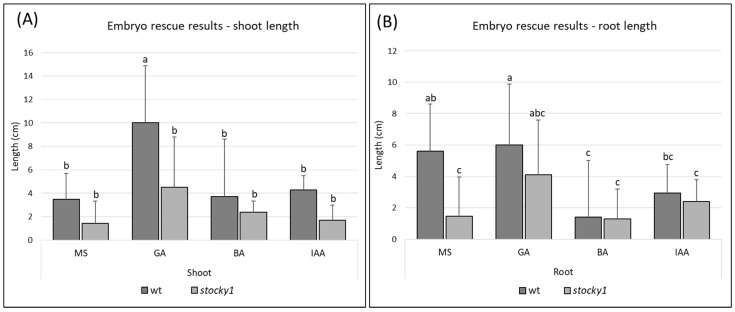
Effect of hormones on *stocky1* growth in embryo-rescue experiment. Immature embryos were collected between 17 and 21 DAP, and were cultured for 10 days before taking root and shoot length measures. The embryos were cultured on MS media; hormones were added at a concentration of 10^−5^ M: (**A**) Graph showing the average length of WT and *stocky1* seedlings shoot 10 days after culture (DAC). (**B**) Graph showing the average length of WT and *stocky1* seedlings root 10 DAC. Error bars represent SD from biological replicates, WT (*n* ≥ 30) and *stocky1* (*n* ≥ 12). Different letters on top of SD indicate a statistically significant difference (*p* < 0.05) obtained with Tukey HSD test after one-way ANOVA.

The phenotype observed in the homozygous *stocky1* mutant, which rapidly leads to plant death, suggested that hormone-related processes are affected by the mutation, thus causing a pleiotropic phenotype. To evaluate this hypothesis, an embryo-rescue experiment was performed, excising an immature embryo from the developing seed at 17/21 DAP, and placing the embryo on Murashige and Skoog (MS) medium supplemented with gibberellic acid (GA), 6-Benzylaminopurine (BA) and Indole-3-acetic acid (IAA). None of the growing media suppressed the lethality of the *stocky1* mutant, and the seedlings were still showing significant defects when growing in all the media supplemented with plant hormones (Figure S1). The lengths of the roots and shoots of the cultured seedlings were measured, showing a consistent reduction in the length of the *stocky1* shoots compared with WT. However, the only significant difference found was between the WT and *stocky1* shoots of the seedlings grown on MS supplemented with GA (Figure 3A).

Regarding root measurement, a statistically significant difference in root length was found between WT and *stocky1* when grown in MS, whereas a statistically significant difference could not be found between WT and *stocky1* roots when the medium was supplemented with hormones (Figure 3B).

### 2.4. Molecular Genetic Analysis of Stocky1

A preliminary physical map position of *stocky1* was obtained through crosses with hyperploid B-A translocation males. The crosses with the TB-A 5S line located the gene on the short arm of chromosome 5. An average of five crosses were performed between plants heterozygous for the mutant and each TB-A (Table 3).

**Figure 4:**
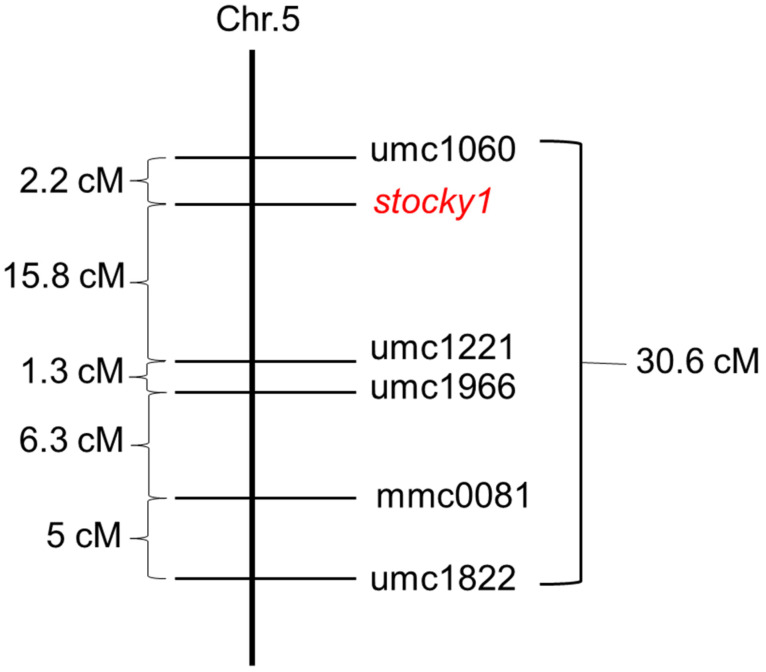
Genetic map of the *stocky1* mutant using SSR molecular markers.

A more refined genetic map position of *stocky1* was achieved by the analysis of simple sequence repeat (SSR) marker distribution in an F3 segregating population consisting of 108 individual seedlings. A polymorphism for the markers umc1060 and umc1221 established the position of the mutant on bin 5.04, at a distance of about 2.2 cm from umc1060 (Figure 4).

### 2.5. Does Stocky1 Encode for a 3-ketoacyl-CoA Synthase (KCS) Gene?

To identify the position of the mutation responsible for the *stocky1* phenotype, we searched the genomic region between the two markers umc1060 and umc1221 for annotated genes involved in the cuticle biosynthesis.

We identified the candidate gene *GRMZM2G569948* encoding a 3-ketoacyl-CoA synthase (KCS) that lies between the two markers at a distance of 22197 kb from umc1060 and at a distance of 10124 kb from umc1221. A set of gene-specific primers were designed to identify possible mutations in the gene *GRMZM2G569948*. However, from the PCR-based analysis, and from the sequencing of the full coding sequence of the gene, no differences between WT and mutant were observed (Figure S2). When the expression level of *GRMZM2G569948* in the shoot of *stocky1* and WT seedlings was tested, no difference was detected (Figure S3).

## 3. Discussion

The survival of the plant in the environment where it germinates is assured by the correctness of the first phases of the seedling development. Rapid and robust emergence allows the plants to compete with neighbors, and to take advantage of favorable conditions. Studying the *des* mutants provides information on different processes of plant development: seed development, SAM establishment and cuticle biosynthesis.

### 3.1. Stocky1 Organs’ Fusion and the Cuticle

In our research, we show a set of newly identified mutants with defects in the seedling development, referred to as *des* mutants. We focused on the analysis of *stocky1*, since it shows delayed seedling development, fusion of leaves TB and loss of the canonical shoot structure. The microscopic analysis and the increased TB permeability in the mutant strongly suggest that the deposition/biosynthesis of cuticle components is impaired in *stocky1*, and this is in line with the observed fusion of the organs. Cases of developmental defects, in particular fusions, have been reported concurrently with the alteration of epidermis-covering materials [7]. For example, the maize mutant *adherent1* (*ad1*), which is deficient in cuticular waxes biosynthesis [11,26], and the maize mutant *fused leaves 1* (*fdl1*), with a defective epicuticular wax deposition, both show organ fusions [16]. In Arabidopsis, there are also many cases where mutants involved in cuticle components’ biosynthesis and deposition show organ fusions: *fiddlehead-1* [27,28,29], *bodyguard* [30], *hothead* [31], *desperado* [32], *lacerata* [33], *cer3/wax2* [34], *lacs1/lacs2* [35].

The *fdl1* mutant shows a very similar phenotype to *stocky1*, with delayed seedling growth and fusions of leaf primordia that lead to the loss of a canonical organization in the shoot [16]. However, the *fdl1* mutant recovers and continues to develop, while *stocky1* is lethal. The *Fdl1* gene is involved in the programmed cell death (PCD) of the apical cells of the coleoptile tip, thus allowing the first leaves to emerge easily. In the stocky mutant, the PCD does not occur, and the coleoptile instead remains green and alive, and is fractured laterally by the emerging leaves that appear curly with glossy regions. In addition, the *adherent1* mutant of maize is viable despite the fusions present in the leaves during the juvenile stage of development and in floral organs (tassel) during the adult stage. It could be that the lethality of our mutant is due to mechanical constraints, since the coleoptile opens normally during the germination, but the second and the other leaves are stuck together inside the conical-shaped structure, blocking further growth.

### 3.2. Genetic Analysis and Gene Candidate

We mapped the *stocky1* mutation on the short arm of chromosome 5 between the molecular markers umc1060 and umc1221. We found that a paralog of *AD1* (*GRMZM2G569948*) is located in the same genomic region. *AD1* encodes for a 3-ketoacyl-CoA synthase (KCS), involved in cuticular waxes biosynthesis: they are composed of a complex mixture of very long-chain fatty acids and their derivatives [36]. A total of 20 *KCS* genes were found in the Arabidopsis genome and 26 in the maize’s genome [37,38]. *GRMZM2G569948* belongs to clade ζ of the *KCS* genes, and it has been classified as *ZmKCS11* [38]. We investigated the possibility that the *stocky1* mutation caused the loss of function of the *ZmKCS11* gene. However, we did not find any differences in the coding sequences of the WT and *stocky1* nor in its expression levels in the developing seedling. These results suggest that ZmKCS11 is functional in the *stocky1* mutant, although we cannot rule out the involvement of post-transcriptional events.

Our preliminary characterization of the *stocky1* mutant shows that the process of cuticle biosynthesis and/or deposition is crucial for correct seedling development and in the prevention of lethal organ fusion. Identifying the gene mutation responsible for the *stocky1* phenotype would be the next research goal. The identification of the *stocky1* mutation could help our understanding of the molecular mechanisms underlying the process of cuticle deposition and biosynthesis during seed development/seedling establishment. The cuticle acts as a diffusion barrier limiting water and solute transport across the apoplast; it protects the plant against chemical and mechanical damage, as well as pest and pathogen attack [39]. For these reasons, research on the cuticle is also relevant for drought tolerance under climate change [25].

## 4. Materials and Methods

### 4.1. Isolation of the Mutants and Genetic Analysis

The des* mutants were originally obtained in 1998 from the M2 progeny of a chemically (ethyl methane sulphonate, EMS) mutagenized population previously used by the group [40]. The genetic background for the mutagenized population was obtained from the cross between two inbred lines, K6, used as female, and a W64A-line pollen mutagenized with EMS. The allelism test was performed on des* mutants using the segregating progeny of selfed heterozygous plants. All des* mutants were crossed in a diallelic test. On average, 20 crosses were performed for each mutant combination. True breeding was determined for each parent: 30–40 kernels from each outcrossed progeny were germinated in soil and scored for the segregation of the defective seedling phenotype.

### 4.2. Gametophytic Selection

To ascertain the presence of gametophytic selection, 40 kernels were taken from the basal and apical sectors of 10 segregating ears, and were used to score for the presence of mutant seedlings after germination in soil.

### 4.3. Fluorescence Microscopy

Large portions of the proximal and distal parts of WT and *stocky1* seedlings were excised at 12 DAS and fixed for 1 h in 4% paraformaldehyde, then washed in PBS and cross-sectioned with a vibratome. Sections of about 20–30 µm were collected and stained for 2 min in Calcofluor White M2R, 0.1%, in water, to facilitate the visualization of leaf structure. The sections were gently washed with distilled water, transferred onto microscope slides and post-stained with Auramine-O 0.1% in 50 mM Tris/HCL at pH 7.2, following the protocol from Nadiminti et al., 2015 [41]. After staining, sections were gently washed with distilled water and mounted with 30% glycerol, and the coverslips sealed with nail polish. A total of 6 slides (3 from proximal and 3 from distal regions) for each seedling were examined by an Olympus BX50 (Tokyo, Japan) equipped with a UV lamp, epipolarization filters and differential interference contrast (DIC), using the exciter filter of 450–480 nm and the barrier filter of 500 nm. Images were recorded with a cooled high-resolution camera (Optika, Ponteranica BG, Italy).

### 4.4. Toluidine Blue Permeability Test

For assessing cuticle integrity, TB tests were performed, as previously described [11,42]. This experiment was conducted on older seedlings compared to the standard procedure used by Liu et al. [11] because the *des* phenotype can be only recognized at 10 DAS. Seedlings at 10 DAS were stained for 5 min in a TB solution (0.05% *w*/*v*) with Tween 20 (0.1% *v*/*v*) and rinsed in tap water. For quantification, the shoots of 3 seedlings were excised, placed in tubes containing 5 mL of 80% ethanol, and incubated overnight at 4 °C in the dark until all dye and chlorophyll had been extracted. Absorbance of the resulting solution was measured at 626 nm and at 430 nm using a spectrophotometer. For each treatment, 3 repeats were performed.

### 4.5. Embryo Culture

For embryo-rescue experiments, several ears from different F3 families, selfed in the field, were taken at 17 and 21 DAP. The ears were sterilized in 5% sodium hypochlorite for 30 min and then rinsed in sterile distilled water. Immature embryos were removed in aseptic conditions and transferred in a Murashige and Skoog (MS) medium (pH 5.6) containing 3% sucrose, solidified with 0.8% agar. Hormones: gibberellic acid (GA_3_), 6-Benzylaminopurine (BA) and Indole-3-acetic acid (IAA) were added at a concentration of 10^−5^ M. Embryos were cultured for 10 days at 25 °C under a long photoperiod (16 h light/8 h dark), and seedling and root elongation were determined.

### 4.6. Mapping of Stocky1

In order to identify the chromosome position of *stocky1*, heterozygous *Stocky1*/*stocky1* females were crossed to a stock carrying the TB-A translocation [43]. The F3 population was obtained after two cycles of selfing of heterozygous plants from the W64A background, introgressed once in the B73 inbred line. Leaves of 2 weeks-old F3 seedlings were used to extract the genomic DNA, following the protocol from Vejlupkova and Fowler, 2003 [44]. Polymerase chain reactions (PCRs) were performed using SSR primer sequences found in the Maize Database. The parameters for the PCRs reactions follow the protocol at //www.maizegdb.org/ssr_protocols 18 January 2022. The PCRs products were visualized on a 4% agarose gel. Recombinant values were converted to map distances through MAPMAKER 3 using Haldane’s mapping function [45]. In total, 108 individual seedlings were used for the genetic map.

### 4.7. Candidate Gene Sequence Analysis

The physical position of the molecular markers umc1060 and umc1221, and of *GRMZM2G569948*, was determined using the Maize GDB, which refers to B73 RefGen_v3 (https://www.maizegdb.org/ accessed on 01 October 2021) [46]. For the sequence analysis of the candidate gene, the genomic DNA was extracted from normal and homozygous *stocky1* seedlings (3 seedlings for both WT and mutant). Two pairs of specific primers indicated in Table S1 were used to perform PCR using the Platinum™ SuperFi™ DNA Polymerase Invitrogen Kit. The reaction mix underwent an initial denaturation step at 94 °C for 2 min, 30 cycles of denaturation at 94 °C for 15 s, annealing at the specific primer temperature for 30 s, and extension at 72 °C for 30 s. Extension at 72 °C for 5 min was performed to complete the reaction. Amplification products were visualized on 1% (*w*/*v*) agarose gels with ethidium bromide staining. The PCR products were sequenced with Sanger method and the sequences were compared with the coding sequence of KCS of the B73 line (ZEAMMB73_Zm00001d016438) obtained from www.maizegdb.org/ on 20 October 2021 using this tool: https://www.ebi.ac.uk/Tools/msa/clustalo/ accessed on 20 October 2021.

### 4.8. RNA, cDNA Preparation, and Quantitative Gene Expression Analysis

RNA was extracted from the shoot of young seedlings at 10 DAS (3 seedlings for both WT and mutant) using the “GeneJET Plant RNA Purification Mini Kit” (ThermoFisher Scientific, Waltham, MA, USA) after powdering the plant tissue with liquid nitrogen. All the extractions were performed following the protocol provided by the manufacturer. RNA samples were long-stored at −80 °C. After extraction, the integrity of RNA was checked on 1% agarose gel. RNA was then treated with DNAse (TURBO DNA-free™ Kit, Invitrogen, Waltham, MA, USA).

RNA was retrotranscripted using the Thermo Fisher Scientific “Maxima First Strand cDNA Synthesis” kit, following the protocol provided by the manufacturer. Specific primers for the housekeeping gene *orange pericarp-1* (*orp-1*) were used to standardize the cDNA concentration. Specific primers were designed to evaluate the gene expression level of the candidate gene *GRMZM2G569948*. The gene-specific primers are listed in Table S1. PCR was performed using the Platinum™ SuperFi™ DNA Polymerase Invitrogen Kit with 34 cycles of amplification. Amplification products were visualized on 1.2% (*w*/*v*) agarose gels with ethidium bromide staining.

## Figures and Tables

**Table 1:** Results for the segregation of mutant phenotypes. m: mutant, Sig: significance (NS = not significant).

Mutants	WT	m	Total	% of Mutants	X^2^ Value for 3:1	Sig.	*P*
des*-4	440	147	587	25.0	0.0006	NS	0.980
*stocky1*	424	117	541	21.6	3.2834	NS	0.700
des*-47	254	71	325	21.8	1.7241	NS	0.189
des*-85	86	24	110	21.8	0.5939	NS	0.441
des*-217	104	38	142	26.8	0.2347	NS	0.6281
des*-256	114	38	152	25.0	0.0000	NS	1

**Table 2:** Results of the complementation test of the des* mutants crossed inter se. (+) and (−) indicate complementation and non-complementation, respectively.

↓	→	des*-4	*stocky1*	des*-47	des*-85	des*-217	des*-256
des*-4		-	+	+	+	+	+
*stocky1*			-	+	+	+	+
des*-47				-	+	+	+
des*-85					-	+	+
des*-217						-	+
des*-256							-

**Table 3:** Result of TB-A crosses with *stocky1*.

TB-A Employed in the Crosses with *Stocky1*	Translocation Uncovering the Mutant
TB 1Sb	+
TB 1La	+
TB 3La-2S	+
TB 3Sb	+
TB 3La	+
TB 5Sc	-
TB 5La	+
TB 7Sc	+
TB 7Lb	+
TB 8Lc	+
TB 9Sb	+
TB 10L19	+

(+) indicates absence of the mutant phenotype in the progeny.

## Data Availability

The data presented in this study are available in article.

## Supplementary Materials

The following are available online at https://www.mdpi.com/article/10.3390/plants11070847/s1, Figure S1: Immature embryos cultured on MS medium supplemented with hormones. Figure S2: Sequence alignment of the coding sequence of the gene GRMZM2G569948. Figure S3: Semi-quantitative RT-PCR analysis of the KCS candidate gene. Table S1: Table of primers.
