# Supplementary material for: Stocky1, a Novel Gene Involved in Maize Seedling Development and Cuticle Integrity
Source: Plants (Basel). 2022 Mar 23;11(7):847. doi: 10.3390/plants11070847 (PMC9003528; doi:10.3390/plants11070847)
Supplement: Supplementary file 1 [file plants-11-00847-s001.zip › Figure S1.pdf]

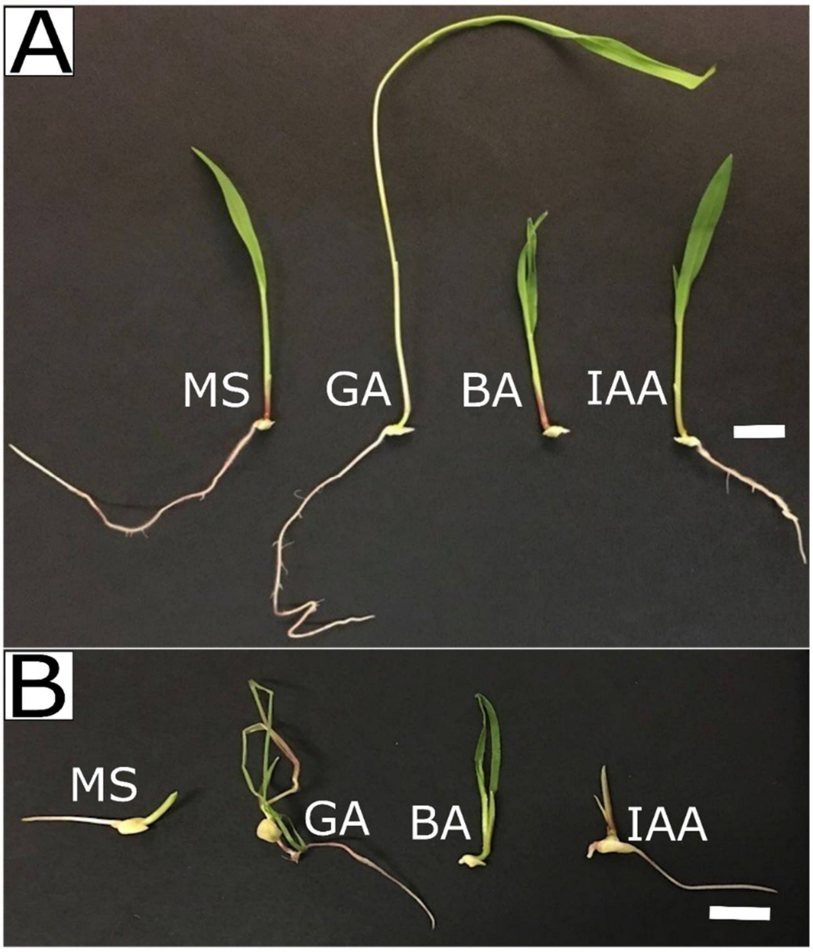

Figure S1. Immature embryos cultured on MS medium supplemented with hormones. Normal seedlings (A) and *stocky1* homozygous seedling (B) after 10 DAC (Days after Culture). The embryos have been grown on MS medium (MS) or on MS medium supplemented with hormones (GA, BA, IAA) at a concentration of 10^{-5} M. Scalebar: 1 cm.
