# Supplementary material for: Stocky1, a Novel Gene Involved in Maize Seedling Development and Cuticle Integrity
Source: Plants (Basel). 2022 Mar 23;11(7):847. doi: 10.3390/plants11070847 (PMC9003528; doi:10.3390/plants11070847)
Supplement: Supplementary file 1 [file plants-11-00847-s001.zip › Figure S2.pdf]

Figure S2. Sequences alignment of the coding sequence of the gene GRMZM2G569948

Alignment of the coding sequence of KCS (GRMZM2G569948) with sequences obtained through Sanger sequencing of PCR based products. 3 primers were used for the sequencing: ZmKCS-3F, ZmKCS-2R, ZmKCS-3R. The sequences of the primers can be found in the Table of primers in the supplementary materials. The coding sequence of KCS (GRMZM2G569948) was downloaded from maizegdb.org ID: ZEAMMB73_Zm00001d016438

```
             ....|....| ....|....| ....|....| ....|....| ....|....|
                     10         20         30         40         50
KcsB73       ATGGAAAACC CGGCGCCGCC GACCAACCCG GCCTCCGCCA CGCCGTCGCC
KCS3Fdes     ATGGAAAACC CGGCGCCGCC GACCAACCCG GCCTCCGCCA CGCCGTCGCC
KCS3FWT      ATGGAAAACC CGGCGCCGCC GACCAACCCG GCCTCCGCCA CGCCGTCGCC
Kcs2Rdes     ---------- ---------- ---------- ---------- ----------
Kcs2RWT      ---------- ---------- ---------- ---------- ----------
KCS3Rdes     ---------- ---------- ---------- ---------- ----------
KCS3RWT      ---------- ---------- ---------- ---------- ----------


             ....|....| ....|....| ....|....| ....|....| ....|....|
                     60         70         80         90        100
KcsB73       GTCGCGGCAG CTGCCGGACT TCCAGCAGTC GGTTCGTCTC AAGTACGTGA
KCS3Fdes     GTCGCGGCAG CTGCCGGACT TCCAGCAGTC GGTTCGTCTC AAGTACGTGA
KCS3FWT      GTCGCGGCAG CTGCCGGACT TCCAGCAGTC GGTTCGTCTC AAGTACGTGA
Kcs2Rdes     ---------- ---------- ---------- ---------- ----------
Kcs2RWT      ---------- ---------- ---------- ---------- ----------
KCS3Rdes     ---------- ---------- ---------- ---------- ----------
KCS3RWT      ---------- ---------- ---------- ---------- ----------


             ....|....| ....|....| ....|....| ....|....| ....|....|
                    110        120        130        140        150
KcsB73       AGCTGGGGTA CCATTACCTC ATCACCCACG GCATGTACCT GCTGCTCACC
KCS3Fdes     AGCTGGGGTA CCATTACCTC ATCACCCACG GCATGTACCT GCTGCTCACC
KCS3FWT      AGCTGGGGTA CCATTACCTC ATCACCCACG GCATGTACCT GCTGCTCACC
Kcs2Rdes     ---------- ---------- ---------- ---------- ----------
Kcs2RWT      ---------- ---------- ---------- ---------- ----------
KCS3Rdes     ---------- ---------- ---------- ---------- ----------
KCS3RWT      ---------- ---------- ---------- ---------- ----------


             ....|....| ....|....| ....|....| ....|....| ....|....|
                    160        170        180        190        200
KcsB73       CCGCTGATGG TGCTCGTGGC CGTGTACCTC TCCACGCTCT CCCCGCGCGA
KCS3Fdes     CCGCTGATGG TGCTCGTGGC CGTGTACCTC TCCACGCTCT CCCCGCGCGA
KCS3FWT      CCGCTGATGG TGCTCGTGGC CGTGTACCTC TCCACGCTCT CCCCGCGCGA
Kcs2Rdes     ---------- ---------- ---------- ---------- ----------
Kcs2RWT      ---------- ---------- ---------- ---------- --CCGCGCGA
KCS3Rdes     ---------- ---------- ---------- ---------- ----------
KCS3RWT      ---------- ---------- ---------- ---------- ----------


             ....|....| ....|....| ....|....| ....|....| ....|....|
                    210        220        230        240        250
KcsB73       CGTCGCCGAC CTGTGGG-CG CACCTCCGCC TCAACCTCAT CTCCGTGCTC
KCS3Fdes     CGTCGCCGAC CTGTGGG-CG CACCTCCGCC TCAACCTCAT CTCCGTGCTG
KCS3FWT      CGTCGCCGAC CTGTGGG-CG CACCTCCGCC TCAACCTCAT CTCCGTGCTC
Kcs2Rdes     ---------- ---------- ---------- ---------- ----------
Kcs2RWT      CGTCGCCGAC CTGTGGGGCG CACCTCCGCC TCAACCTCAT CTCCGTGCTC
KCS3Rdes     ---------- ---------- ---------- ---------- ----------
KCS3RWT      ---------- ---------- ---------- ---------- ----------


             ....|....| ....|....| ....|....| ....|....| ....|....|
                    260        270        280        290        300
KcsB73       GCCTGCTCCA CGCTCCTCGT CTTCCTCGGC ACGGCCTACT TCCTCACCCG
KCS3Fdes     GCCTGCTCCA CGCTCCTCGT CTTCCTCGGC ACGGCCTACT TCCTCACCCG
KCS3FWT      GCCTGCTCCA CGCTCCTCGT CTTCCTCGGC ACGGCCTACT TCCTCACCCG
Kcs2Rdes     ---------- CGCTCCTCGT CTTCCTCGGC ACGGCCTACT TCCTCACCCG
Kcs2RWT      GCCTGCTCCA CGCTCCTCGT CTTCCTCGGC ACGGCCTACT TCCTCACCCG
KCS3Rdes     ---------- ---------- ---------- ---------- ----------
KCS3RWT      ---------- ---------- ---------- ---------- ----------
```

```
              ....|....| ....|....| ....|....| ....|....| ....|....|
                     310        320        330        340        350
KcsB73        CCCGCGG-CC CGTGTACCTG GTCGACTTCG CCTGCTACAA GCCCGGGCCG
KCS3Fdes      CCCGCGG-CC CGTGTACCTG GTCGACTTCG CCTGCTACAA GCCCGGGCCG
KCS3FWT       CCCGCGG-CC CGTGTACCTG GTCGACTTCG CCTGCTACAA GCCCGGGCCG
Kcs2Rdes      CCCGCGGGCC CGTGTACCTG GTCGACTTCG CCTGCTACAA GCCCGGGCCG
Kcs2RWT       CCCGCGG-CC CGTGTACCTG GTCGACTTCG CCTGCTACAA GCCCGGGCCG
KCS3Rdes      ---------- ---------- ---------- ---------- ----------
KCS3RWT       ---------- ---------- ---------- ---------- ----------

              ....|....| ....|....| ....|....| ....|....| ....|....|
                     360        370        380        390        400
KcsB73        GAGCGCCGGT GCACGCGCGA CACCTTCATG CGCTGCTCCA GGCTCACCGG
KCS3Fdes      GAGCGCCGGT GCACGCGCGA CACCTTCATG CGCTGCTCCA GGCTCACCGG
KCS3FWT       GAGCGCCGGT GCACGCGCGA CACCTTCATG CGCTGCTCCA GGCTCACCGG
Kcs2Rdes      GAGCGTCGGT GCACGCGCGA CACCTTCATG CGCTGCTCCA GGCTCACCGG
Kcs2RWT       GAGCGCCGGT GCACGCGCGA CACCTTCATG CGCTGCTCCA GGCTCACCGG
KCS3Rdes      ---------- ---------- ---------- ---------- ----------
KCS3RWT       ---------- ---------- ---------- ---------- ----------

              ....|....| ....|....| ....|....| ....|....| ....|....|
                     410        420        430        440        450
KcsB73        CTGCTTCACC GACGCCAGCC TCGAGTTCCA GCGCAAGATC CTGGAGCGCT
KCS3Fdes      CTGCTTCACC GACGCCAGCC TCGAGTTCCA GCGCAAGATC CTGGAGCGCT
KCS3FWT       CTGCTTCACC GACGCCAGCC TCGAGTTCCA GCGCAAGATC CTGGAGCGCT
Kcs2Rdes      CTGCTTCACT GACGCCAGCC TCGAGTTCCA GCGCAAGATC CTGGAGCGCT
Kcs2RWT       CTGCTTCACC GACGCCAGCC TCGAGTTCCA GCGCAAGATC CTGGAGCGCT
KCS3Rdes      ---------- ---------- ---------- ---------- ----------
KCS3RWT       ---------- ---------- ---------- ---------- ----------

              ....|....| ....|....| ....|....| ....|....| ....|....|
                     460        470        480        490        500
KcsB73        CGGGGCTGGG CGAGGACACG TACCTTCCCC CCGCCGTCAC GCGGGTGCCG
KCS3Fdes      CGGGGCTGGG CGAGGACACG TACCTTCCCC CCGCCGTCAC GCGGGTGCCG
KCS3FWT       CGGGGCTGGG CGAGGACACG TACCTTCCCC CCGCCGTCAC GCGGGTGCCG
Kcs2Rdes      CGGGGCTGGG CGAGGACACG TACCTTCCCC CCGCCGTCAC GCGGGTGCCG
Kcs2RWT       CGGGGCTGGG CGAGGACACG TACCTTCCCC CCGCCGTCAC GCGGGTGCCG
KCS3Rdes      ---------- ---------- ---------- ---------- ----------
KCS3RWT       ---------- ---------- ---------- ---------- ----------

              ....|....| ....|....| ....|....| ....|....| ....|....|
                     510        520        530        540        550
KcsB73        CCCAACCCGT CCATGGACGA GGCGCGCGCG GAGGCGCGGG AGGTCATGTT
KCS3Fdes      CCCAACCCGT CCATGGACGA GGCGCGCGCG GAGGCGCGGG AGGTCATGTT
KCS3FWT       CCCAACCCGT CCATGGACGA GGCGCGCGCG GAGGCGCGGG AGGTCATGTT
Kcs2Rdes      CCCAACCCGT CCATGGACGA GGCGCGCGCG GAGGCGCGGG AGGTCATGTT
Kcs2RWT       CCCAACCCGT CCATGGACGA GGCGCGCGCG GAGGCGCGGG AGGTCATGTT
KCS3Rdes      ---------- ---------- ---------- ---------- ----------
KCS3RWT       ---------- ---------- ---------- ---------- ----------

              ....|....| ....|....| ....|....| ....|....| ....|....|
                     560        570        580        590        600
KcsB73        CGGCGCCGTT GACGAGCTGC TCGCCAAGAC GGGGGTCAAG CCCAAAGACA
KCS3Fdes      CGGCGCCGTT GACGAGCTGC TCGCCAAGAC GGGGGTCAAG CCCAAAGACA
KCS3FWT       CGGCGCCGTT GACGAGCTGC TCGCCAAGAC GGGGGTCAAG CCCAAAGACA
Kcs2Rdes      CGGCGCCGTT GACGAGCTGC TCGCCAAGAC GGGGGTCAAG CCCAAAGACA
Kcs2RWT       CGGCGCCGTT GACGAGCTGC TCGCCAAGAC GGGGGTCAAG CCCAAAGACA
KCS3Rdes      ---------- ---------- ---------- ---------- ----------
KCS3RWT       ---------- ---------- ---------- ---------- ----------

              ....|....| ....|....| ....|....| ....|....| ....|....|
                     610        620        630        640        650
KcsB73        TCGGGATCCT CGTGGTGAAC TGCAGCCTGT TCAACCCGAC GCCGTCGCTG
KCS3Fdes      TCGGGATCCT CGTGGTGAAC TGCAGCCTGT TCAACCCGAC GCCGTCGCTG
KCS3FWT       TCGGGATCCT CGTGGTGAAC TGCAGCCTGT TCAACCCGAC GCCGTCGCTG
```

```
Kcs2Rdes     TCGGGATCCT CGTGGTGAAC TGCAGCCTGT TCAACCCGAC GCCGTCGCTG
Kcs2RWT      TCGGGATCCT CGTGGTGAAC TGCAGCCTGT TCAACCCGAC GCCGTCGCTG
KCS3Rdes     ---------- ---------- ---------- ---------- ----------
KCS3RWT      ---------- ---------- ---------- ---------- ----------


             ....|....| ....|....| ....|....| ....|....| ....|....|
                    660        670        680        690        700
KcsB73       TCGGCCATGG TGGTCAACCA CTACAAGCTG CGCGGGAACG TGGTGAGCTA
KCS3Fdes     TCGGCCATGG TGGTCAACCA CTACAAGCTG CGCGGGAACG TGGTGAGCTA
KCS3FWT      TCGGCCATGG TGGTCAACCA CTACAAGCTG CGCGGGAACG TGGTGAGCTA
Kcs2Rdes     TCGGCCATGG TGGTCAACCA CTACAAGCTG CGCGGGAACG TGGTGAGCTA
Kcs2RWT      TCGGCCATGG TGGTCAACCA CTACAAGCTG CGCGGGAACG TGGTGAGCTA
KCS3Rdes     ---------- ---------- ---------- ---------- ----------
KCS3RWT      ---------- ---------- ---------- ---------- ----------


             ....|....| ....|....| ....|....| ....|....| ....|....|
                    710        720        730        740        750
KcsB73       CAACCTCGGC GGGATGGGGT GCAGCGCC-G GGCTGCTGTC CGTGGACCTC
KCS3Fdes     CAACCTCGGC GGGATGGGGT GCAGCGCCCG GGCTGCTGTC CGTGGACCTC
KCS3FWT      CAACCTCGGC GGGATGGGGT GCAGCGCC-G GGCTGCTGTC CGTGGACCTC
Kcs2Rdes     CAACCTCGGC GGGATGGGGT GCAGCGCC-G GGCTGCTGTC CGTGGACCTC
Kcs2RWT      CAACCTCGGC GGGATGGGGT GCAGCGCC-G GGCTGCTGTC CGTGGACCTC
KCS3Rdes     ---------- ---------- ---------- ---------- -----~~~~~
KCS3RWT      ---------- ---------- ---------- -------~~~ ~~--~~~~~~


             ....|....| ....|....| ....|....| ....|....| ....|....|
                    760        770        780        790        800
KcsB73       GCCAAGGACC TGCTGCAGAC GCACCCGGGG TCGTACGCGC TGGTCATCAG
KCS3Fdes     ---------- ---------- ---------- ---------- ----------
KCS3FWT      GCCAAGGACC TGCTGCAGAC GCACCCGGGG TCGTACGCGC TGGTCATCAG
Kcs2Rdes     GCCAAGGACC TGCTGCAGAC GCACCCGGGG TCGTACGCGC TGGTCATCAG
Kcs2RWT      GCCAAGGACC TGCTGCAGAC GCACCCGGGG TCGTACGCGC TGGTCATCAG
KCS3Rdes     ~~~~~~~~~~ ~~~~~~~~~~ ~~~~~~~~~~ TCGTACGCGC TGGTCATCAG
KCS3RWT      ~~~~~~~~~~ ~~~~~~~~~~ ~~~~~~~~~~ TCGTACGCGC TGGTCATCAG


             ....|....| ....|....| ....|....| ....|....| ....|....|
                    810        820        830        840        850
KcsB73       CACGGAGAAC ATCACGCTCA ACTGGTACTC GGGGAACGAC CGCTCCAAGC
KCS3Fdes     ---------- ---------- ---------- ---------- ----------
KCS3FWT      C--------- ---------- ---------- ---------- ----------
Kcs2Rdes     CACGGAGAAC ATCACGCTCA ACTGGTACTC GGGGAACGAC CGCTCCAAGC
Kcs2RWT      CACGGAGAAC ATCACGCTCA ACTGGTACTC GGGGAACGAC CGCTCCAAGC
KCS3Rdes     CACGGAGAAC ATCACGCTCA ACTGGTACTC GGGGAACGAC CGCTCCAAGC
KCS3RWT      CACGGAGAAC ATCACGCTCA ACTGGTACTC GGGGAACGAC CGCTCCAAGC


             ....|....| ....|....| ....|....| ....|....| ....|....|
                    860        870        880        890        900
KcsB73       TGGTGTCCAA CTGCCTGTTC CGGATGGGCG GCGCCGCGGT GCTGCTCTCG
KCS3Fdes     ---------- ---------- ---------- ---------- ----------
KCS3FWT      ---------- ---------- ---------- ---------- ----------
Kcs2Rdes     TGGTGTCCAA CTGCCTGTTC CGGATGGGCG GCGCCGCGGT GCTGCTCTCG
Kcs2RWT      TGGTGTCCAA CTGCCTGTTC CGGATGGGCG GCGCCGCGGT GCTGCTCTCG
KCS3Rdes     TGGTGTCCAA CTGCCTGTTC CGGATGGGCG GCGCCGCGGT GCTGCTCTCG
KCS3RWT      TGGTGTCCAA CTGCCTGTTC CGGATGGGCG GCGCCGCGGT GCTGCTCTCG


             ....|....| ....|....| ....|....| ....|....| ....|....|
                    910        920        930        940        950
KcsB73       AATCGGCGGT CCGACCGGCG GCGGGCCAAG TACGAGCTGG TGCACACGGT
KCS3Fdes     ---------- ---------- ---------- ---------- ----------
KCS3FWT      ---------- ---------- ---------- ---------- ----------
Kcs2Rdes     AATCGGCGGT CCGACCGGCG GCGGGCCAAG TACGAGCTGG TGCACACGGT
Kcs2RWT      AATCGGCGGT CCGACCGGCG GCGGGCCAAG TACGAGCTGG TGCACACGGT
KCS3Rdes     AATCGGCGGT CCGACCGGCG GCGGGCCAAG TACGAGCTGG TGCACACGGT
KCS3RWT      AATCGGCGGT CCGACCGGCG GCGGGCCAAG TACGAGCTGG TGCACACGGT
```

```
                 ....|....| ....|....| ....|....| ....|....| ....|....|
                        960        970        980        990       1000
KcsB73           GCGCACGCAC AAGGGCGCGG ACGACCGGTG CTTCGGCTGC GTGACGCAGG
KCS3Fdes         ---------- ---------- ---------- ---------- ----------
KCS3FWT          ---------- ---------- ---------- ---------- ----------
Kcs2Rdes         GCGCACGCAC AAGGGCGCGG ACGACCGGTG CTTCGGCTGC GTGACGCAGG
Kcs2RWT          GCGCACGCAC AAGGGCGCGG ACGACCGGTG CTTCGGCTGC GTGACGCAGG
KCS3Rdes         GCGCACGCAC AAGGGCGCGG ACGACCGGTG CTTCGGCTGC GTGACGCAGG
KCS3RWT          GCGCACGCAC AAGGGCGCGG ACGACCGGTG CTTCGGCTGC GTGACGCAGG

                 ....|....| ....|....| ....|....| ....|....| ....|....|
                       1010       1020       1030       1040       1050
KcsB73           AGGAGGACGG CGAGGGCGTC GTGGGCGTGT CGCTGTCGAG GGACCTGATG
KCS3Fdes         ---------- ---------- ---------- ---------- ----------
KCS3FWT          ---------- ---------- ---------- ---------- ----------
Kcs2Rdes         AGGAGGACGG CGAGGGCGTC GTGGGCGTGT CGCTGTCGAG GGACCTGATG
Kcs2RWT          AGGAGGACGG CGAGGGCGTC GTGGGCGTGT CGCTGTCGAG GGACCTGATG
KCS3Rdes         AGGAGGACGG CGAGGGCGTC GTGGGCGTGT CGCTGTCGAG GGACCTGATG
KCS3RWT          AGGAGGACGG CGAGGGCGTC GTGGGCGTGT CGCTGTCGAG GGACCTGATG

                 ....|....| ....|....| ....|....| ....|....| ....|....|
                       1060       1070       1080       1090       1100
KcsB73           GCGGTGGCGG GCGACGCGCT CAAGACGAAC ATAACAACGC TGGGCCCGCT
KCS3Fdes         ---------- ---------- ---------- ---------- ----------
KCS3FWT          ---------- ---------- ---------- ---------- ----------
Kcs2Rdes         GCGGTGGCGG GCGACGCGCT CAAGACGAAC ATAACAACGC TGGGCCCGCT
Kcs2RWT          GCGGTGGCGG GCGACGCGCT CAAGACGAAC ATAACAACGC TGGGCCCGCT
KCS3Rdes         GCGGTGGCGG GCGACGCGCT CAAGACGAAC ATAACAACGC TGGGCCCGCT
KCS3RWT          GCGGTGGCGG GCGACGCGCT CAAGACGAAC ATAACAACGC TGGGCCCGCT

                 ....|....| ....|....| ....|....| ....|....| ....|....|
                       1110       1120       1130       1140       1150
KcsB73           GGTGCTGCCG CTGTCGGAGC AGCTGCTGTT CATGGCGACG CTGGTCGCCA
KCS3Fdes         ---------- ---------- ---------- ---------- ----------
KCS3FWT          ---------- ---------- ---------- ---------- ----------
Kcs2Rdes         GGTGCTGCCG CTGTCGGAGC AGCTGCTGTT ---------- ----------
Kcs2RWT          GGTGCTGCCG CTGTCGGAGC AGCTGCTGTT ---------- ----------
KCS3Rdes         GGTGCTGCCG CTGTCGGAGC AGCTGCTGTT CATGGCGACG CTGGTCGCCA
KCS3RWT          GGTGCTGCCG CTGTCGGAGC AGCTGCTGTT CATGGCGACG CTGGTCGCCA

                 ....|....| ....|....| ....|....| ....|....| ....|....|
                       1160       1170       1180       1190       1200
KcsB73           AGAAGCTGCT CAAGATGAAG AAGGTGAAGC CGTACATCCC GGATTTCAAA
KCS3Fdes         ---------- ---------- ---------- ---------- ----------
KCS3FWT          ---------- ---------- ---------- ---------- ----------
Kcs2Rdes         ---------- ---------- ---------- ---------- ----------
Kcs2RWT          ---------- ---------- ---------- ---------- ----------
KCS3Rdes         AGAAGCTGCT CAAGATGAAG AAGGTGAAGC CGTACATCCC GGATTTCAAA
KCS3RWT          AGAAGCTGCT CAAGATGAAG AAGGTGAAGC CGTACATCCC GGATTTCAAA

                 ....|....| ....|....| ....|....| ....|....| ....|....|
                       1210       1220       1230       1240       1250
KcsB73           CTGGCGTTCG AGCACTTCTG CATCCACGCC GGCGGCCGTG CCGTGCTGGA
KCS3Fdes         ---------- ---------- ---------- ---------- ----------
KCS3FWT          ---------- ---------- ---------- ---------- ----------
Kcs2Rdes         ---------- ---------- ---------- ---------- ----------
Kcs2RWT          ---------- ---------- ---------- ---------- ----------
KCS3Rdes         CTGGCGTTCG AGCACTTCTG CATCCACGCC GGCGGCCGTG CCGTGCTGGA
KCS3RWT          CTGGCGTTCG AGCACTTCTG CATCCACGCC GGCGGCCGTG CCGTGCTGGA

                 ....|....| ....|....| ....|....| ....|....| ....|....|
                       1260       1270       1280       1290       1300
KcsB73           CGAGCTGGAG AGCAACCTGG CGCTCACGGA TTGGCACATG GAGCCGTCGC
KCS3Fdes         ---------- ---------- ---------- ---------- ----------
KCS3FWT          ---------- ---------- ---------- ---------- ----------
Kcs2Rdes         ---------- ---------- ---------- ---------- ----------
```

```
Kcs2RWT      ---------- ---------- ---------- ---------- ----------
KCS3Rdes     CGAGCTGGAG AGCAACCTGG CGCTCACGGA TTGGCACATG GAGCCGTCGC
KCS3RWT      CGAGCTGGAG AGCAACCTGG CGCTCACGGA TTGGCACATG GAGCCGTCGC


             ....|....| ....|....| ....|....| ....|....| ....|....|
                   1310       1320       1330       1340       1350
KcsB73       GGATGACGCT GCACCGGTTC GGGAACACGT CCAGCAGCTC GCTCTGGTAC
KCS3Fdes     ---------- ---------- ---------- ---------- ----------
KCS3FWT      ---------- ---------- ---------- ---------- ----------
Kcs2Rdes     ---------- ---------- ---------- ---------- ----------
Kcs2RWT      ---------- ---------- ---------- ---------- ----------
KCS3Rdes     GGATGACGCT GCACCGGTTC GGGAACACGT CCAGCAGCTC GCTCTGGTAC
KCS3RWT      GGATGACGCT GCACCGGTTC GGGAACACGT CCAGCAGCTC GCTCTGGTAC


             ....|....| ....|....| ....|....| ....|....| ....|....|
                   1360       1370       1380       1390       1400
KcsB73       GAGCTGGCCT ACAGCGAGGC CAAGGGGAGG ATCCGGCGCC GCCACCGGGT
KCS3Fdes     ---------- ---------- ---------- ---------- ----------
KCS3FWT      ---------- ---------- ---------- ---------- ----------
Kcs2Rdes     ---------- ---------- ---------- ---------- ----------
Kcs2RWT      ---------- ---------- ---------- ---------- ----------
KCS3Rdes     GAGCTGGCCT ACAGCGAGGC CAAGGGGAGG ATCCGGCGCC GCCACCGGGT
KCS3RWT      GAGCTGGCCT ACAGCGAGGC CAAGGGGAGG ATCCGGCGCC GCCACCGGGT


             ....|....| ....|....| ....|....| ....|....| ....|....|
                   1410       1420       1430       1440       1450
KcsB73       GTGGCAGATC GCGTTCGGGT CAGGGTTCAA GTGCAACAGC GCCGTGTGGA
KCS3Fdes     ---------- ---------- ---------- ---------- ----------
KCS3FWT      ---------- ---------- ---------- ---------- ----------
Kcs2Rdes     ---------- ---------- ---------- ---------- ----------
Kcs2RWT      ---------- ---------- ---------- ---------- ----------
KCS3Rdes     GTGGCAGATC GCGTTCGGGT CAGGGTTCAA GTGCAACAGC GCCGTGTGGA
KCS3RWT      GTGGCAGATC GCGTTCGGGT CAGGGTTCAA GTGCAACAGC GCCGTGTGGA


             ....|....| ....|....| ....|....| ....|....| ....|....|
                   1460       1470       1480       1490       1500
KcsB73       GGGCGCTCCG GTCGGTGAAC CCGGCGGAGG AGACGAACCC GTGGATGGAC
KCS3Fdes     ---------- ---------- ---------- ---------- ----------
KCS3FWT      ---------- ---------- ---------- ---------- ----------
Kcs2Rdes     ---------- ---------- ---------- ---------- ----------
Kcs2RWT      ---------- ---------- ---------- ---------- ----------
KCS3Rdes     GGGCGCTCCG GTCGGTGAAC CCGGCGGAGG AGACGAACCC GTGGATGGAC
KCS3RWT      GGGCGCTCCG GTCGGTGAAC CCGGCGGAGG AGACGAACCC GTGGATGGAC


             ....|....| ....|....| ....|....| ....|....| ....|....|
                   1510       1520       1530       1540       1550
KcsB73       GAGATCGACA GGTTTCCCGT GGATGTTCCC AAGGTCTCTA AGGTTTCAAG
KCS3Fdes     ---------- ---------- ---------- ---------- ----------
KCS3FWT      ---------- ---------- ---------- ---------- ----------
Kcs2Rdes     ---------- ---------- ---------- ---~~~~~~~ ~~~~~~~~~~
Kcs2RWT      ---------- ---------- ---------- ---~~~~~~~ ~~~~~~~~~~
KCS3Rdes     GAGATCGACA GGTTTCCCGT GGATGTTCCC AAGGTCTCTA AGGTTTCAAG
KCS3RWT      GAGATCGACA GGTTTCCCGT GGATGTTCCC AAGGTCTCTA AGGTTTCAAG


             ....|....| ....|....| ....|....| ....|....| ....|....|
                   1560       1570       1580       1590       1600
KcsB73       CGACTGA--- ---------- ---------- ---------- ----------
KCS3Fdes     ---------- ---------- ---------- ---------- ----------
KCS3FWT      ---------- ---------- ---------- ---------- ----------
Kcs2Rdes     ~~~~~~~--- ---------- ---------- ---------- ----------
Kcs2RWT      ~~~~~~~--- ---------- ---------- ---------- ----------
KCS3Rdes     CGACTGAAAT GCATTGCTAG TTGCGTGAAT GATGGAGCTT GCAATTGTTT
KCS3RWT      CGACTGAAAT GCATTGCTAG TTGCGTGAAT GATGGAGCTT GCAATTGTTT


             ....|....| ....|....| ....|....| ....|....| ....|....|
```

```
                 1610       1620       1630       1640       1650
KcsB73      ---------- ---------- ---------- ---------- ----------
KCS3Fdes    ---------- ---------- ---------- ---------- ----------
KCS3FWT     ---------- ---------- ---------- ---------- ----------
Kcs2Rdes    ---------- ---------- ---------- ---------- ----------
Kcs2RWT     ---------- ---------- ---------- ---------- ----------
KCS3Rdes    GGATAGAAGA ---------- ---------- ---------- ----------
KCS3RWT     GGATAGAAGA TCTGGTGGAA TTTTTCATAG TTCAT----- ----------
```
