# Supplementary material for: Stocky1, a Novel Gene Involved in Maize Seedling Development and Cuticle Integrity
Source: Plants (Basel). 2022 Mar 23;11(7):847. doi: 10.3390/plants11070847 (PMC9003528; doi:10.3390/plants11070847)
Supplement: Supplementary file 1 [file plants-11-00847-s001.zip › Figure S3.pdf]

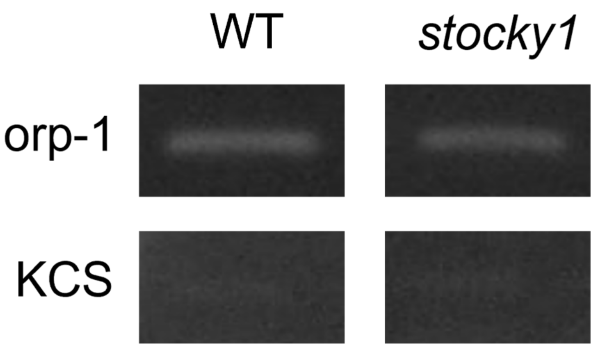

Figure S3. Semi quantitative RT-PCR analysis of the KCS candidate gene.
In the first lane are shown the bands of the orp-1 gene, the house-keeping gene which has been used to normalize the quantity of the cDNA between wild type and the mutant *stocky1.* The bands shown in the second lane are referring to the KCS candidate gene, and the amplification is weak in both the cDNA obtained from wild- type and the des*-19 seedlings.
