# Supplementary material for: Stocky1, a Novel Gene Involved in Maize Seedling Development and Cuticle Integrity
Source: Plants (Basel). 2022 Mar 23;11(7):847. doi: 10.3390/plants11070847 (PMC9003528; doi:10.3390/plants11070847)
Supplement: Supplementary file 1 [file plants-11-00847-s001.zip › Table S1.pdf]

Table S1. Table of primers.

Name	Sequence 5'-3'	Information
ZmKCS-4F	CATGGTGGTCAACCACTACAA	used for semi-quantitative PCR
ZmKCS-4R	TGATGTTCTCCGTGCTGATG	used for semi-quantitative PCR
ZmKCS-3F	CTTCAGCTAGCCACATCTCTC	used for sequencing KCS coding sequence
ZmKCS-3R	GTCGATCGCCTTTCCTATCTC	used for sequencing KCS coding sequence
ZmKCS-1F	CGCACGTAGGACAACTGATAA	used for sequencing KCS coding sequence
ZmKCS-2R	GATGTACGGCTTCACCTTCTT	used for sequencing KCS coding sequence
ORP1-F	AAGGACGTGCACACCGC	used for semi-quantitative PCR
ORP1-R	CAGATACAGAACAACAACTC	used for semi-quantitative PCR
